# Intravital imaging with two-photon microscopy reveals cellular dynamics in the ischeamia-reperfused rat heart

**DOI:** 10.1038/s41598-018-34295-w

**Published:** 2018-10-30

**Authors:** Ryohei Matsuura, Shigeru Miyagawa, Satsuki Fukushima, Takasumi Goto, Akima Harada, Yuri Shimozaki, Kazumasa Yamaki, Sho Sanami, Junichi Kikuta, Masaru Ishii, Yoshiki Sawa

**Affiliations:** 1 Department of Cardiovascular Surgery, Osaka University Graduate School of Medicine, Osaka, Japan; 2 Department of Immunology and Cell Biology, Osaka University Graduate School of Medicine, Osaka, Japan; 3 Research and Development Division for Advanced Technology, Research and Development Center, Dai Nippon Printing Co., Ltd., Tokyo, Japan

## Abstract

Recent advances in intravital microscopy have provided insight into dynamic biological events at the cellular level in both healthy and pathological tissue. However, real-time *in vivo* cellular imaging of the beating heart has not been fully established, mainly due to the difficulty of obtaining clear images through cycles of cardiac and respiratory motion. Here we report the successful recording of clear *in vivo* moving images of the beating rat heart by two-photon microscopy facilitated by cardiothoracic surgery and a novel cardiac stabiliser. Subcellular dynamics of the major cardiac components including the myocardium and its subcellular structures (i.e., nuclei and myofibrils) and mitochondrial distribution in cardiac myocytes were visualised for 4–5 h in green fluorescent protein-expressing transgenic Lewis rats at 15 frames/s. We also observed ischaemia/reperfusion (I/R) injury-induced suppression of the contraction/relaxation cycle and the consequent increase in cell permeability and leukocyte accumulation in cardiac tissue. I/R injury was induced in other transgenic mouse lines to further clarify the biological events in cardiac tissue. This imaging system can serve as an alternative modality for real time monitoring in animal models and cardiological drug screening, and can contribute to the development of more effective treatments for cardiac diseases.

## Introduction

Intravital confocal and two-photon microscopy have been used in combination with fluorescence molecular imaging probes in cancer research, immunology, and neuroscience to investigate biological processes at the cellular level in living organisms^1–5^. Two-photon microscopy has unique advantages over conventional single-photon confocal microscopy^6,7^: the excitation beam can achieve deeper penetration, and two-photon excitation markedly reduces overall photobleaching and photodamage, thereby extending the viability of biological specimens during long-term imaging. These properties are particularly useful for monitoring the function of cardiomyocytes in the living heart.

Optical microscopy can potentially be used to assess cardiomyocyte structure and function in rodent models^7^, but the application of intravital techniques for imaging the beating heart has been limited by motion from cardiac contraction and respiration; consequently, most studies have used non-contracting Langendorf heart preparations^8–15^ or transplanted heart models^16^ that do not allow investigation of cardiomyocyte biology under physiological conditions. A few studies have achieved intravital imaging of orthotopic hearts at relatively modest spatial and temporal resolutions.

A system for *in vivo* subcellular imaging of the mouse heart using a unique tissue stabiliser and acquisition gating was developed^17–19^, but requires cardiac pacing, gating, and retrospective image reconstruction. There have been no reports of real-time *in vivo* imaging of cardiac tissue dynamics under ischaemia/reperfusion (I/R) conditions by intravital microscopy at subcellular resolution^20–24^. Motion compensation and cardiac tissue stabilisation are two major challenges for the use of intravital microscopy for imaging beating hearts in physiological and pathological states.

**Figure 1:**
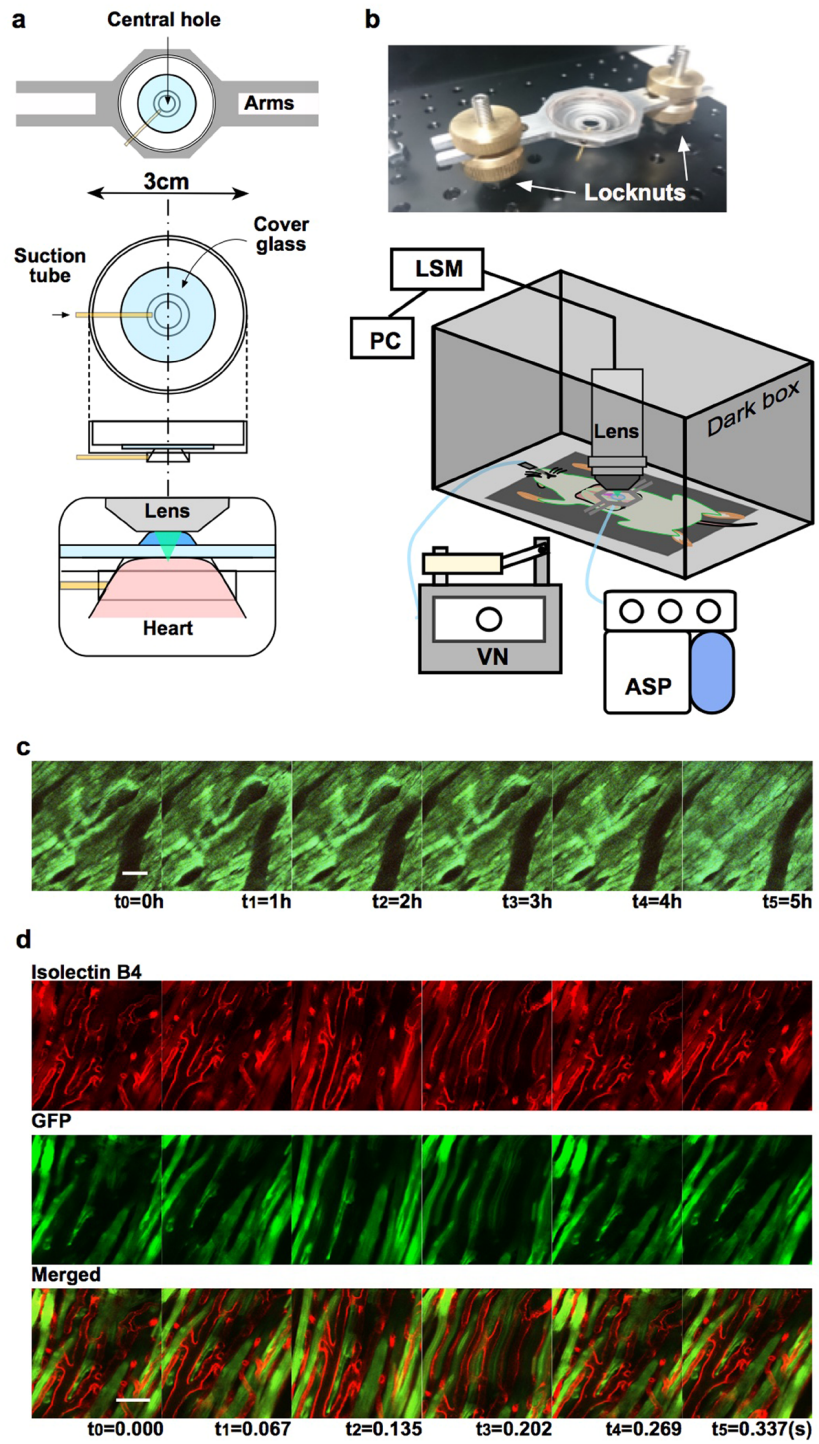
Experimental setup and real-time *in vivo* imaging of normal heart beating. (**a**) Stabiliser design: a central hole with a diameter of 8 mm diameter and a depth of 1 mm was cut and then completely countersunk at a 50° angle to the plane of the stabiliser. The hole was covered with a round coverglass. A suction tube could access the chamber through the lateral wall of the central hole—which protruded slightly above the plane of the stabiliser—and could attach to the heart surface by suction, as shown in the enlarged side view. (**b**) After optimising the height from the steel plate, the stabiliser was fixed by interpolating the bilateral arms between locknuts on two threaded pillars protruding from the plate. The equipment included a water-dipping objective lens, ventilator (VN), aspirator (ASP), laser-scanning microscope (LSM), and personal computer (PC). (**c**) Longitudinal time-lapse imaging of the beating heart in a GFP rat. The cardiomyocyte structure was labelled with GFP. No photobleaching occurred during recording at 15 frames/s (Supplementary Fig. 1). Scale bar, 100 µm. Panels (t_0_–t_5_) show representative frames recorded every hour. (**d**) Visualisation of the dynamic motion of capillaries between cardiac myocytes. The endothelium of the microvasculature was stained with isolectin B4 (red, left panels); cardiomyocytes were labelled with GFP (green, middle panels). Scale bar, 50 µm (Supplementary Video 1). Panels (t_0_–t_5_) show representative frames recorded consecutively over a single cardiac cycle (15 frames/s).

We developed a two-photon microscopy system equipped with a custom-built cardiac tissue stabiliser (Fig. 1a) facilitated by cardiothoracic surgery for imaging a beating heart *in vivo* and successfully recorded clear, real-time, hours-long videos of beating rat hearts with and without regional cardiac I/R. Using fluorescent reporters that provide molecular pathway-specific readouts, this system achieved subcellular spatial resolution and millisecond temporal resolution. Intravital imaging allowed visualisation of the contraction and relaxation of myofibril structures of individual cardiac myocytes, distribution of mitochondria in myocytes, and blood flow in capillaries under non-ischaemic conditions. In addition, dynamic changes in each component under ischaemic conditions and in reperfused cardiac tissue were documented in videos and still images.

## Results

Transgenic Lewis rats ubiquitously expressing green fluorescent protein (GFP)^25,26^ were placed under general anaesthesia with mechanical ventilation. The anterior thoracic wall was completely resected to expose the left ventricle (LV). Fluorescent dextran and/or isolectin B4 (conjugated with Texas Red and Cascade Blue)^27,28^ were intravenously injected to visualise blood flow and/or the endothelium, respectively. Mitochondrial membranes were labelled by intravenous injection of tetramethylrhodamine, ethyl ester, perchlorate (TMRE)^21,22^. The coverglass attached to a custom-made suction device was placed over the anterior LV wall of rats in a dark box with the intravital microscope system, which was composed of a two-photon upright microscope equipped with a 25× water-dipping objective lens (Fig. 1b).

**Figure 2:**
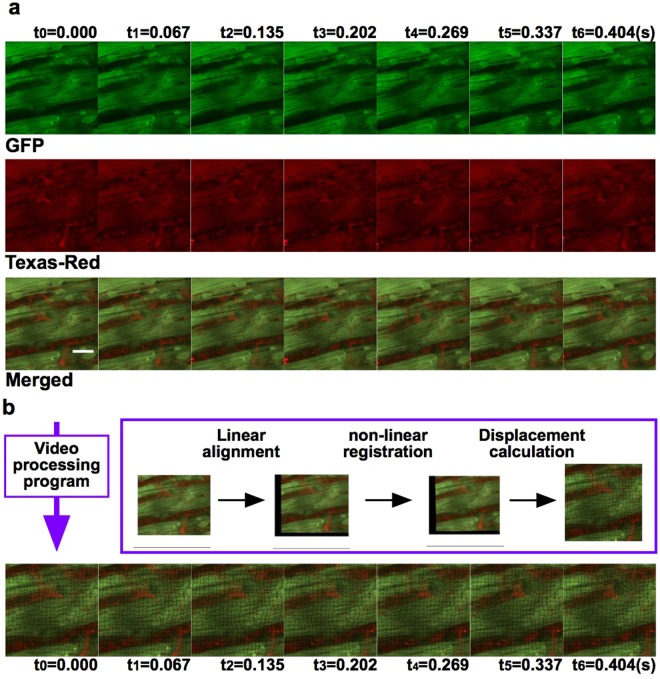
Real-time *in vivo* imaging of cardiomyocytes and blood flow; and video-processing program. (**a**) Visualisation of regular motion of intercellular connections between adjacent cardiac myocytes and blood flow through capillaries and venules. Green, GFP; red, red-labelled dextran. Scale bar, 20 µm (Supplementary Video 2). (**b**) Analysis of the motion of normal cardiomyocytes facilitated by stabilisation of the beating heart by linear alignment and non-linear registration, followed by measurement of inter-pixel distance between reference points on a grid in the first frame and in each subsequent frame (Supplementary Video 3). Each image series (t_0_–t_6_) in Fig. 2 shows the movement over a period of approximately one heartbeat.

The myocardium and its subcellular structures (i.e., nuclei and myofibrils) were stabilised for 5 h and recorded at regular intervals for a total of 5 minutes of irradiation with no photobleaching (Fig. 1c, Supplementary Fig. 1 and Video 1). The dynamic motion of capillaries between cardiac myocytes was visualised by injecting Texas Red-labelled isolectin, which is selective for terminal alpha-D-galactosamine end groups expressed in the cell membrane of endothelial cells^27,28^ (Fig. 1d and Supplementary Video 2). The synchronised contractions of adjacent cardiac myocytes were also visualised. Notably, blood flow through the capillaries (Fig. 2a and Supplementary Video 3) and venules was clearly visible following the Texas Red-labelled dextran injection. The velocity of leukocytes was also successfully recorded by tracking their movement across frames (Supplementary Fig. 2).

The motion analysis process for cardiomyocytes comprised three steps (Fig. 2b). The first step was ordinary image registration for large-scale stabilisation, which compensated for movements in XY translation and XY rotation. The second step was non-linear registration to establish local correspondence. The final step was displacement calculation. We measured the relative movement of sample points through frame-by-frame mapping of corresponding points (Fig. 2b and Supplementary Video 4).

**Figure 3:**
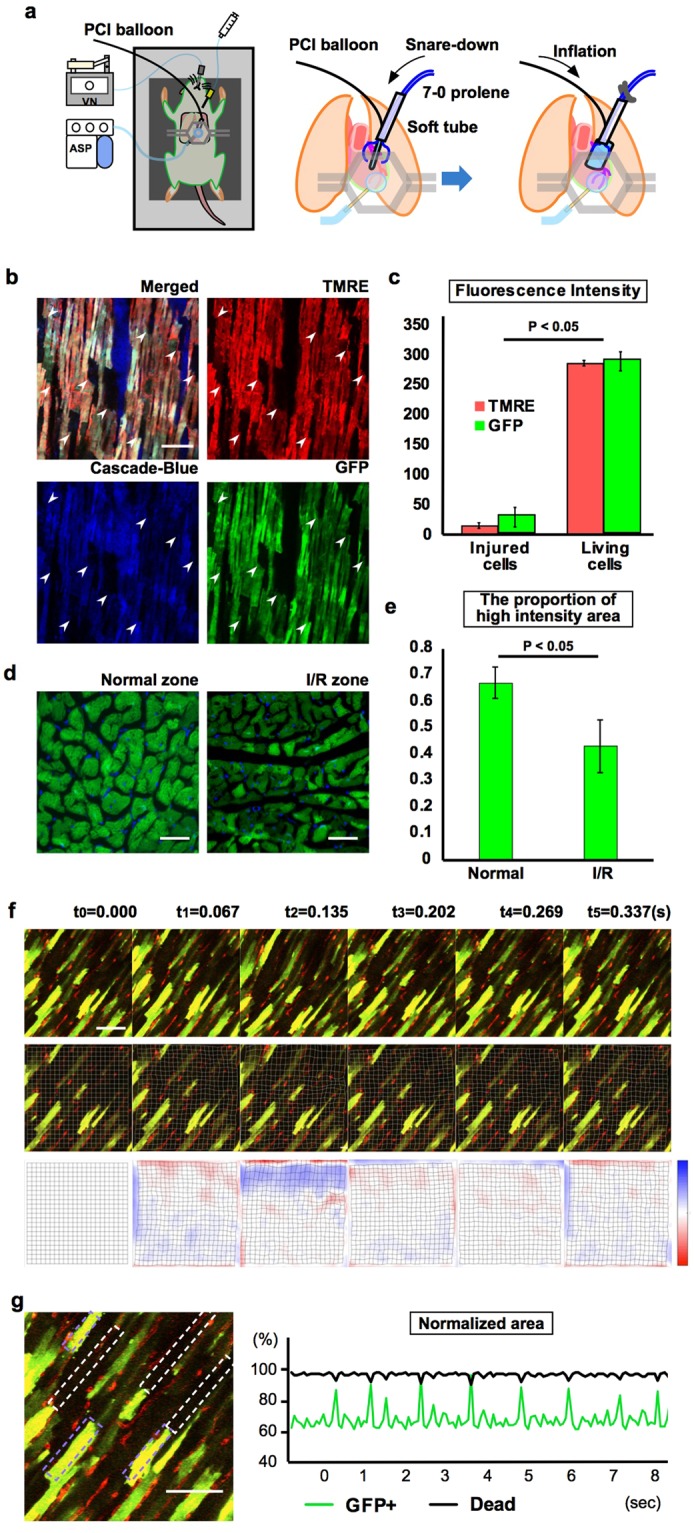
Motion analysis of injured cardiac myocytes. (**a**) Experimental setup. A balloon catheter for percutaneous coronary intervention (PCI balloon) was placed at the base of the left atrial appendage and interposed in the loop of the polypropylene suture. The suture was snared without compromising blood flow through the artery. Remote manual inflation of the balloon induced occlusion of the left anterior descending artery to generate broad anterior LV wall ischaemia. (**b**) Visualisation of the motion of injured cardiac myocytes (Supplementary Video 5). Delineated high- and low-intensity fluorescent patches in the injured heart were clearly observed. Low-intensity patches corresponded to injured cardiomyocytes (white arrowheads). Green, GFP; blue, blue-labelled dextran; red, TMRE. Scale bar, 100 µm. (**c**) TMRE-stained and unstained areas were considered as living and dead tissue, respectively. GFP intensity was strongly correlated with TMRE staining, with a sharp distinction observed between living and dead areas (n = 3). Scale bar, 100 µm. (**d**) Fluorescence analysis of cardiomyocytes labelled with GFP (green) in fixed cardiac tissue; nuclei were counterstained with DAPI (blue). The I/R region had a large area of cardiomyocytes with low-intensity fluorescent patches. Scale bar, 50 µm. (**e**) Proportion of high-intensity fluorescent area relative to total cardiomyocyte area (n = 3). (**f**) Measurement of the amplitude of real-time movement of the reference grid allowed visualisation of relative motion within the image (Supplementary Videos 6 and 7). Scale bar, 100 µm. Red and blue colours show contraction and relaxation, respectively, in the lower image series. (**g**) Quantitative analysis of differences in patch size fluctuations between living and injured cells, with the largest difference set to 100%. Green, GFP; blue, blue-labelled dextran; red, TMRE. Scale bar, 100 µm.

We developed an I/R model in which a 2.5 × 15-mm balloon catheter used for percutaneous coronary intervention was interposed in the loop of the polypropylene suture placed around the left anterior descending coronary artery at the base of the left atrial appendage. The suture tails were snared into a tube without compromising blood flow through the artery (Fig. 3a). This allowed remote manual inflation of the balloon to induce occlusion of the left anterior descending artery, resulting in broad ischaemia in the anterior LV wall. After 15 min ischaemia and 15 min reperfusion, we observed patchy loss of GFP in sarcomeres and mitochondrial membranes of cardiac myocytes labelled with TMRE around the ischaemic zone (Fig. 3b and Supplementary Video 5), which was indicative of mitochondria-related deterioration of the cellular environment associated with cell death^21,22,29^ (Fig. 3c). The presence of low-intensity patches was confirmed in the I/R model by histological analysis (Fig. 3d,e).

Motion analysis of the videos revealed differences between normal and injured cardiac myocytes. We analysed changes in area based on GFP fluorescence intensity (Fig. 3f and Supplementary Video 6). GFP-positive regions showed stronger cyclic contractions than dead regions (Fig. 3g and Video 7). The cycles appeared to be in opposite phases, with areas of high fluorescence expanding while those with low fluorescence were contracting, and *vice versa*.

**Figure 4:**
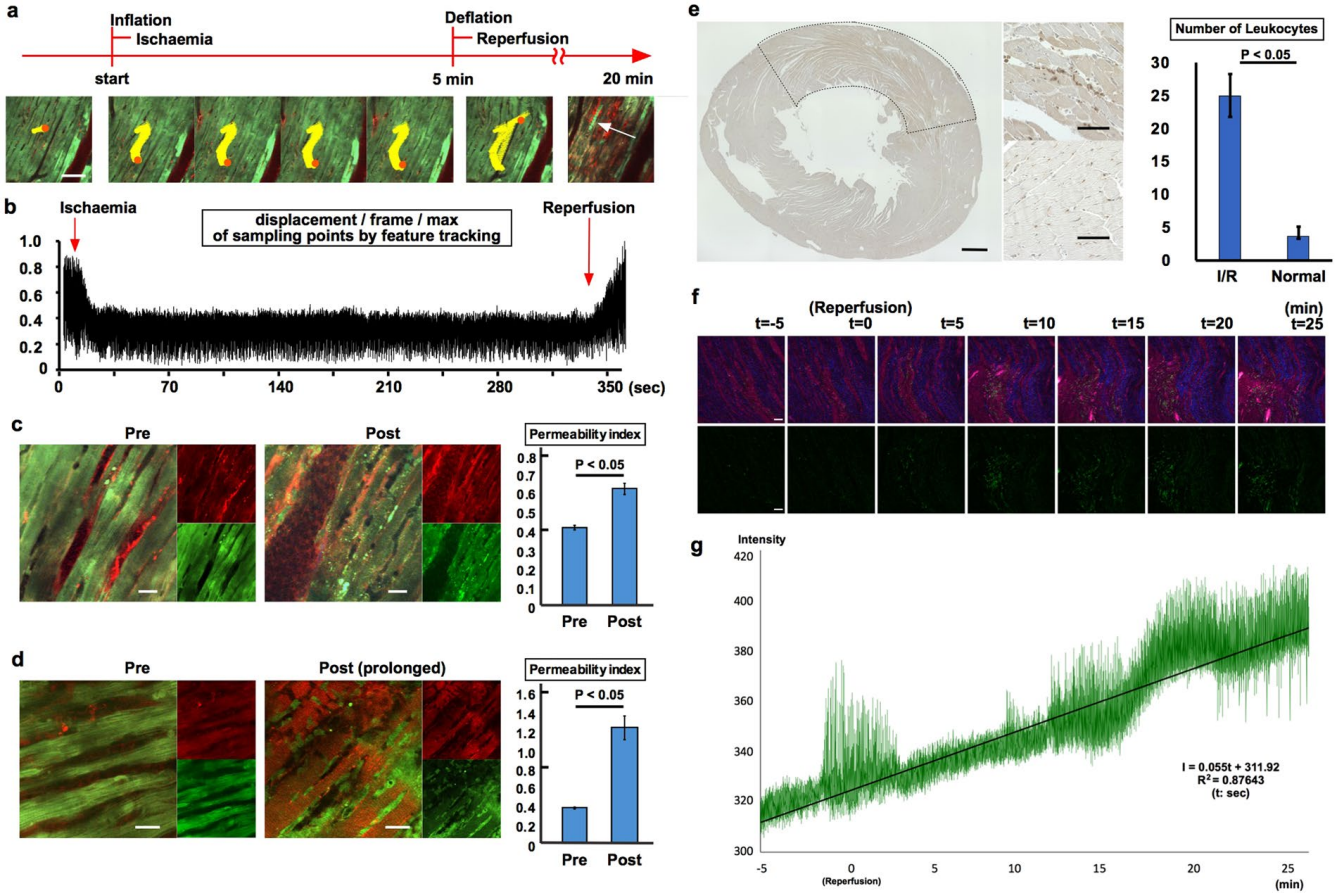
I/R set-up and quantitative analysis of imaging data. (**a**) Supplementary Video 8 showing the moments of ischaemia and reperfusion. Feature tracking of an arbitrary point (marked in orange) was used to determine the amplitude of the beating heart (Supplementary Video 9); the yellow jagged line is the path traced by the arbitrary point. The white arrow indicates accumulated leukocytes. Scale bar, 100 µm. (**b**) Displacement amplitude per frame of an arbitrary point (maximum displacement = 1.0). Amplitude decreased during ischaemia and recovered after reperfusion. (**c**) Permeability index. The index was defined as relative intensity of red fluorescence (I_green_ = 1.0) of muscle areas divided by that of blood vessel areas. The permeability index was higher 15 min post-reperfusion (Post, Supplementary Video 10) than pre-reperfusion (Pre) using the 5-min ischaemia protocol. Green, GFP; red, red-labelled dextran. Scale bar, 20 µm. (n = 3) (**d**) Permeability index determined using the prolonged I/R protocol, which resulted in a greater increase in the post-reperfusion index, indicating greater penetration of red-labelled dextran into muscle tissues. Micro-capillaries were blocked by leukocytes adhering to the wall following 1 h of ischaemia and 1 h of reperfusion (Supplementary Video 11). Green, GFP; red, red-labelled dextran. Scale bar, 20 µm. (n = 3) (**e**) Immunohistological analysis with anti-myeloperoxidase antibody. The region surrounded by the dotted line is the ischemia reperfusion area. The graph shows the average number of leukocytes in regions of interest. (n = 3) (**f**) Panels (upper; merge, Texas red, lower; green) show representative frames recorded every 5 minutes (Supplementary Video 12). [t = 0] Indicates the timing of reperfusion. Upper panels; merge; Red, red-labelled dextran; Green, GFP; Blue, Second Harmonic Generation. Lower panels; Green, GFP. Scale bar, 50 µm. (**g**) The graph shows the change of green fluorescence intensity in the whole region of interest over time in Video 12. The equation shown in the figure is linearly approximated.

It was also possible to record continuous movements and changes in the myocardium over an extended period of time in the I/R model; a gradual overall reduction in the contraction and relaxation of cardiac myocytes was observed in the target region (Fig. 4a and Supplementary Video 8). Reperfusion following 5 min of ischaemia resulted in a rapid recovery of blood flow in the venules accompanied by re-dilatation of capillaries and an increase in amplitude of the beating heart (Fig. 4b, Supplementary Video 9). Notably, Texas Red-labelled dextran penetrated cardiac myocytes after I/R (15 min) and GFP fluorescence intensity was not restored, indicating intracellular acidosis^29^ (Fig. 4c, Supplementary Video 10).

A 1-h reperfusion following 1 h of ischaemia induced accumulation of static GFP-positive leukocytes in the area of interest, with concomitant loss of GFP signal in cardiac myocytes (Fig. 4d, Supplementary Video 11). Thus, cellular-level imaging provided a detailed view of the no-reflow phenomenon^30–32^. Immunolabelling for myeloperoxidase revealed leukocyte infiltration into the reperfusion area (Fig. 4e). In bone marrow transplantation rats in which transplanted bone marrow cells are labelled with GFP, white blood cells invaded the reperfusion area following I/R (Fig. 4f,g and Supplementary Video 12).

## Discussion

Myocardial ischaemia remains a common cause of morbidity and mortality worldwide, with the rates predicted to increase over the coming decades^33^. Cardiomyocytes are the fundamental unit of myocardial function; as such, clarifying the molecular basis for their responses to stress—and the failure thereof—in both individual cells and in the whole tissue is important for establishing appropriate disease models and designing effective targeted therapies.

Methods of macrostabilisation are essential and various techniques have been employed for this purpose including affixing the heart with sutures^34^, compressing the heart with a coverslip^16^, bonding the heart with a mechanical stabiliser^17^, or using a suction-based device^35,36^. Although such methods can facilitate very-low-resolution cardiac imaging (e.g., of microvasculature, cellular recruitment, and flow), they cannot achieve the temporal or spatial resolution necessary for subcellular imaging of cardiomyocytes throughout the cardiac cycle. Gating algorithms for segmented microscopy have been applied to overcome residual motion artifacts^17^, but their retrospective nature and low temporal resolution limit their utility.

We developed a cardiac surface stabiliser for the adult rat anterior LV wall inspired by the suction-based device used for anastomosis in off-pump coronary artery bypass graft surgery^37^ that achieved greater stability than what has previously been reported^16,17,34–36^. The relatively simple design is predicted to have wide applicability even in complex models such as the I/R model reported here, thereby enabling the real-time observation of changes in myocardial tissues. This is an improvement over previously reported techniques, which required not only electrocardiogram-triggered gating and respiratory synchronisation but also post-processing to reconstruct the data into a dynamic image^17–19^, and were predicated on the assumption that the same phenomena occur over every period of the cardiac cycle. Improved time resolution has rendered reconstruction unnecessary and has allowed leukocyte velocity to be more accurately recorded. It is also possible to capture changes occurring over several seconds—for example movements within the period of a single myocardial contraction/relaxation cycle following administration of a cardiotonic agent such as catecholamine or in cases of arrhythmia. In terms of the structure of the stabiliser, the distance from the focal plane and the cover glass was kept constant, but any information on movement along the z axis was missing. It is necessary to assume that significant deviation occurs only in the horizontal directions (imaging plane) to achieve adequate frame rate for observing subcycle dynamics.

We also completely resected the anterior thoracic wall to allow adjustment of the stabiliser and the upright microscope during monitoring. The basic principles of two-photon microscopy presented herein are applicable to other experimental animals including mice, guinea pigs, or domestic pigs, and has potential applications in humans as a next-generation cardiac diagnostic imaging tool. An upright microscope for imaging through a temporary chest opening is necessary for clinical purposes. Our imaging method is not suitable for time-lapse imaging in its present configuration since it involves removal of the chest wall; procedural and technical refinement of the setup is required.

The two-photon laser has low toxicity and better penetration through tissue than with a confocal microscope, allowing continuous visualisation of deeper tissue without photobleaching. In the present work, the tissue depth for imaging was limited to within 100 µm from the cardiac surface (Supplementary Fig. 3 and Video 13). This is the maximum imaging depth of fluorescence microscopy, but it is nonetheless sufficient to access most major coronary vessels as well as capillaries and the myocardium. Additionally, there is still room for improvement in three-dimensional image reconstruction with respect to the frame rate (Supplementary Fig. 4 and Video 14). A transgenic rat strain ubiquitously expressing GFP was useful for observing sarcomere structure without labelling, and enabled visualisation of a phenomenon that was presumably intracellular acidosis in cardiac myocytes.

The unavoidably large magnitude of heart movements has made it difficult to monitor cellular-level dynamics in injured myocardium. For instance, our understanding of I/R injury at various stages of cardiovascular disease is limited due to the lack of methodologies for phenotyping heart function at the subcellular scale *in vivo* and in real time. For the first time, the heterogeneity of cardiomyocyte death and leukocyte accumulation have been directly and comprehensively observed *in vivo* in real time using our microscopy system. It is thought that during myocardial I/R, cardiac cell death, calcium overload, and mitochondrial permeability transition pore opening occur, resulting in the generation of reactive oxygen species^38–40^. Leukocytes and neutrophils are also said to infiltrate the ischaemic area during reperfusion and secrete various serine proteases^41^. Further development of fluorescent reporters with minimal toxicity would enable more detailed investigation of a variety of other phenomena in cardiac tissue.

## Methods

### GFP rats

Adult male and female CAG/GFP transgenic Lewis rats (weighing 250–400 g; Shimizu Laboratory Supplies, Kyoto, Japan) were used for all studies. Transgenic Lewis rats ubiquitously expressing the CAG/GFP marker gene were generated according to a previously described protocol^25,26^.

### Generation of GFP bone marrow transplantation model

Bone marrow cells were isolated from 9- to 11-week old male Wister rats ubiquitously expressing enhanced GFP. Recipients were 5-week old male Wistar rats irradiated with 10 Gy X-ray radiation. The following day, each irradiated rat was injected with 3 × 10^7^ bone marrow cells from GFP transgenic rats. Animal studies were approved by the Ethics Review Committee for Animal Experimentation of Osaka University School of Medicine and conformed to the Guide for the Care and Use of Laboratory Animals published by the U.S. National Institutes of Health.

### Surgical preparation and stabiliser setup

Rats were placed under general anaesthesia with isoflurane (Pfizer, New York, NY, USA) via an endotracheally intubated 18-gauge plastic tube with mechanical ventilation (3 ml tidal volume 50 times/min) (Shinano, Tokyo, Japan; Cat. No. SN-480-7) on a stainless steel plate. The forefeet and tube were fixed at the plate. The anterior chest wall was excised by careful haemostasis using bipolar scissors (Force FX-CS, E4051CT; Valleylab, Denver, USA). After incision of the pericardium, the heart was exposed and an injection line was placed in the cervical vein to inject fluorescence dye. The anterior wall of the heart was affixed by gentle suction to the central hole of the stabiliser. The heart with the stabiliser was then carefully adjusted for observation, without compromising the heartbeat or blood flow. After optimising the height, the stabiliser was fixed by interpolating its bilateral legs between the locknuts. Suction pressure was maintained during imaging with an aspirator. This stabilisation approach was based on the surgical practice of human coronary artery surgery under beating-heart conditions^20^.

### Two-photon intravital heart imaging

The intravital microscope system was composed of a two-photon microscope (A1-MP; Nikon, Tokyo, Japan) with a laser (Chameleon Vision II Ti:Sapphire; Coherent, Santa Clara, CA, USA) tuned to 800–880 nm and an upright microscope equipped with a 25× water immersion objective lens (CFI Apo 25 × W MP; Nikon). For cardiac tissue imaging, the above-described plate-stabiliser setup was placed on a two-axis translation stage under the objective lens in a temperature-controlled dark box. The stage could be translated along both horizontal axes to move the rat and stabiliser together until the objective lens was aligned with the central hole of the stabiliser. After adjusting the objective lens through the Binoscope, the beating heart tissue was viewed *in vivo* via the central hole. After closing the black-out curtain and switching the light path from the Binoscope to the front, live imaging was initiated. The focal plane in the tissue was adjusted remotely by controlling the distance of the objective lens. Fluorescent signals were detected through band-pass emission filters at 492 nm (for blue-labelled dextran), 525/50 nm (for GFP), and 629/53 nm (for 70-kDa red-labelled dextran, isolectin B4, and TMRE).

The system acquired three-colour images (512 × 512 pixels) at 15 frames/s. The images were displayed in real time on a computer monitor and saved to a hard disk. For three-dimensional videos, four sequential image stacks were acquired at 3-μm z-spacing to cover a volume of 84.85 × 84.85 × 45.00 μm (width × height × depth). The time resolution was 1 min. Raw data were processed with NIS-Elements software (Nikon).

### I/R protocol

As shown in Fig. 3a, a suture thread for snares (7-0 Prolene; Johnson and Johnson, New Brunswick, NJ, USA) was looped around the left anterior descending coronary artery at the base of the left atrial appendage. A long and narrow balloon for percutaneous coronary intervention (2.5 × 15 mm, Tazuna; Teruma, Tokyo, Japan) was interposed in the loop of the suture, threaded through a firm plastic tube (1-mm diameter), and snared down gently without moving the balloon or compromising blood flow through the artery. Remote manual inflation of the balloon induced occlusion of the left anterior descending artery, resulting in broad anterior LV wall ischaemia. The duration of I/R was 15 min/15 min (Fig. 3b–g), 30 min/30 min (Fig. 3f), and 1 h/1 h (Fig. 4c–e).

### Histochemistry, immunostaining, and immunofluorescence labeling

The ventricles were fixed in formalin, embedded in paraffin, and cut into 5-μm sections on a microtome for histological analysis. The sections were labelled with polyclonal anti-myeloperoxidase antibody (1:100, ab9535; Abcam, Cambridge, UK) and visualised with the Labeled Streptavidin–Biotin kit (K0690, Dako, Glostrup, Denmark), an automated immunostaining system.

### Cardiac histomorphometry (image data analysis)

Cardiac morphometry was carried out by measuring the area of cardiomyocytes; however, the visual field of the acquired video was displaced by the heartbeat and the image was distorted by the movement of the tissue during the scan period of each frame. To quantify myocardial motion from the videos, we extracted the motion of the cardiomyocytes by removing the other movements as described below using an in-house program developed with Matlab software.

#### Linear alignment

To remove the movement of the heart, the video was processed by shifting the position and orientation of each frame image relative to a reference frame as global spatial alignment^42^. A frame was first selected as a positioning reference. In this example, the first frame was taken as the reference frame. We selected two characteristic regions (a_0_, b_0_) in the reference frame image and stored the coordinates of the centre of each region (pa_0_, pb_0_). Both points needed to be identifiable in all other frames and the coordinates were stored in subsequent frames (pa_1_, pa_2_, … pa_n_; pb_1_, pb_2_, … pb_n_). All other frames were then adjusted (translational/rotational movement only) in the following manner: pa_n_ was moved to pa_0_ for each image. The angle of the straight line connecting pa_n_ and pb_n_ was the same as that connecting pa_0_ and pb_0_ by rotating the image around pa_n_. With this procedure, we stabilised the image of the heart in the video.

#### Non-linear registration

To remove distortion caused by tissue motion resulting from myocardial contractions, the video was processed using a non-rigid matching technique for local spatial alignment in 2D image space^42^. Image transformation was performed to minimise deviations from the reference image while maintaining smoothness representing a spline-based FFD; this parameter was determined only to remove tissue motion in this step. A video was produced in which only the motion of the myocardium was preserved while distortion attributable to myocardial contractions was removed.

#### Displacement calculations

To measure displacement due to myocardial contractions, the displacement of points in the reference image was computed using a non-rigid matching technique with a different parameter. The reference image was selected from the results of the non-linear registration step, and the parameter representing smoothness was determined for the cell contraction. This process produced a video suitable for measuring myocardial contraction amplitudes.

### Fluorescence intensity and permeability index

Fluorescence intensity was calculated from the spectra of each filter using NIS-Elements. The permeability index was defined as relative red intensity (I_green_ = 1.0) of muscle tissues divided by that of blood vessels.

### Statistical analysis

Quantitative values are presented as mean ± SD. P values were calculated with the Student’s t test. Differences were considered statistically significant at P < 0.05.

## Electronic supplementary material

Supplementary Information

Video 1

Video 2

Video 3

Video 4

Video 5

Video 6

Video 7

Video 8

Video 9

Video 10

Video 11

Video 12

Video 13

Video 14
