# Supplementary material for: Intravital imaging with two-photon microscopy reveals cellular dynamics in the ischeamia-reperfused rat heart
Source: Sci Rep. 2018 Oct 30;8:15991. doi: 10.1038/s41598-018-34295-w (PMC6207786; doi:10.1038/s41598-018-34295-w)
Supplement: Supplementary file 1 — Supplementary Information [file 41598_2018_34295_MOESM1_ESM.pdf]

Scientific Reports

Supplementary Information

Intravital imaging with two-photon microscopy reveals cellular dynamics in the ischeamia-reperfused rat heart

Ryohei Matsuura[1], Shigeru Miyagawa[1], Satsuki Fukushima[1], Takasumi Goto[1], Akima Harada[1], Yuri Shimozaki[3], Kazumasa Yamaki[3], Sho Sanami[3], Junichi Kikuta[2], Masaru Ishii[2], Yoshiki Sawa[1]

[1]Department of Cardiovascular Surgery, Osaka University Graduate School of Medicine, Osaka, Japan

[2]Department of Immunology and Cell Biology, Osaka University Graduate School of Medicine, Osaka, Japan

[3]Research and Development Division for Advanced Technology, Research and Development Center, Dai Nippon Printing Co., Ltd., Tokyo, Japan

Figure 1

(a) **Longitudinal two-photon imaging of the beating heart**. Time-measurement of GFP intensity in Longitudinal two-photon imaging of the beating heart in the GFP Lewis rat stabilized for 5 hours and recorded at regular intervals for a total of 5 minutes of irradiation. The graph indicates the recording was performed without photobleaching. (see Video 1)

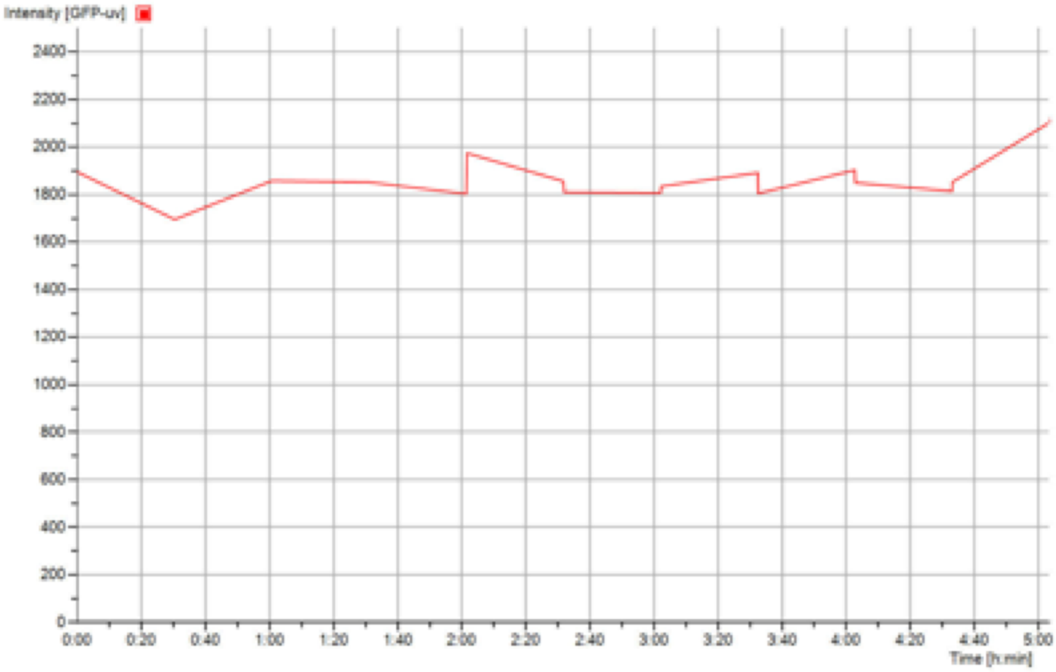

(b) **Images of normal GFP rat heart and intensities of each channel.** Images of normal GFP rat heart were collected using two photon microscopy, without administrating any staining solutions at 840 nm (Video 1). Scale bar, 50 µm. The graph shows that intensities in the GFP normal cardiac tissue were achieved for green signal and green bleed-through in the red and blue channel.

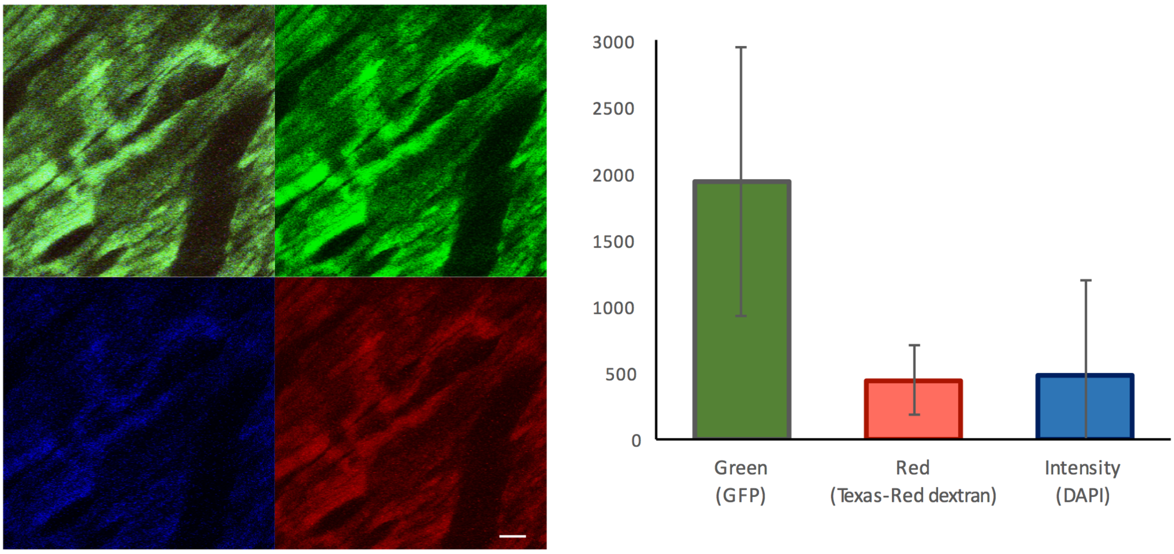

Figure 2

The velocity measurement of leukocyte flowing in Ischeamia/Reperfusion zone on the Video 10. Four leukocytes labelled by GFP were identified and tracked frame by frame. The numbers plotted on each graph are the elapsed times (msec). White arrows in each sequential images are one of the leukocytes.

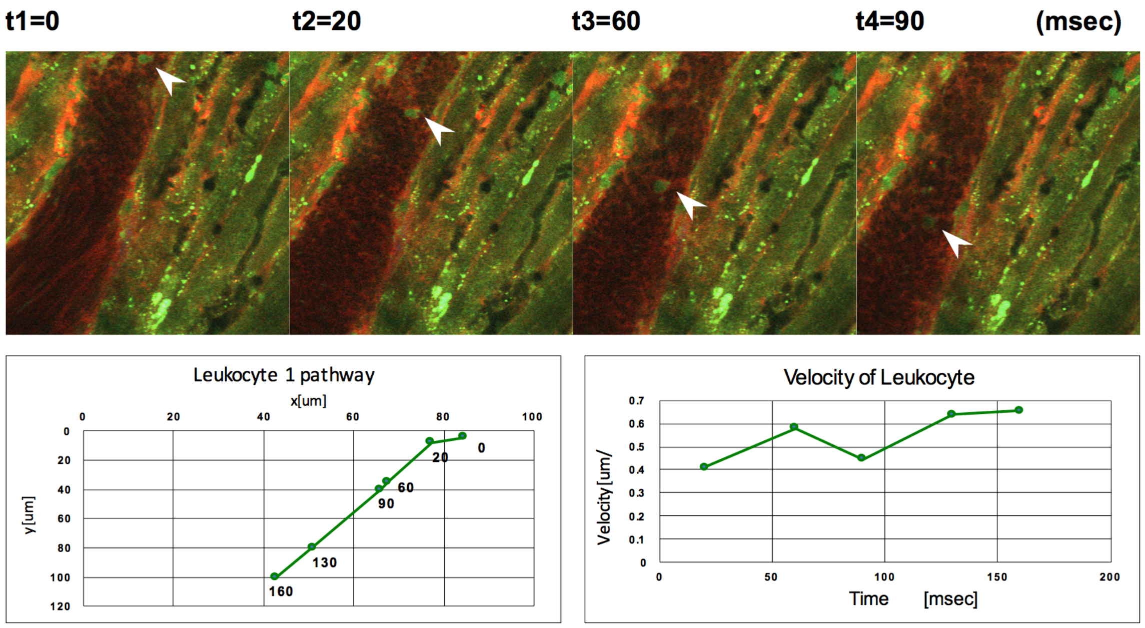

Figure 3, Video 13

Myocardial tissue stained with TMRE in the arrested heart of GFP Lewis rat. The right and bottom images are each cross-sectional views of the myocardial tissue. Scale bar: 100 µm. Z-stack was used as experimental loop for output timing parameter.

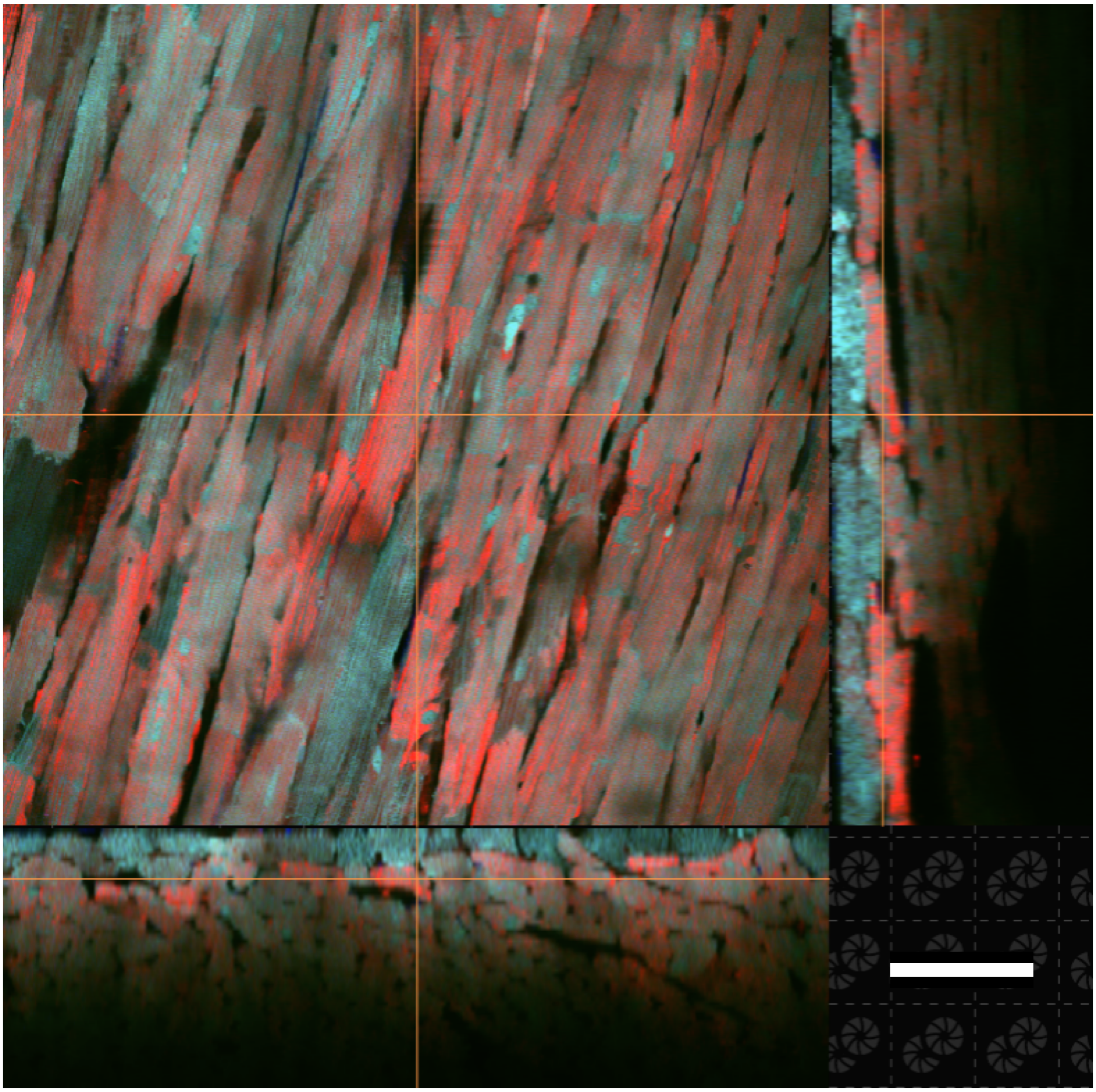

Figure 4, Video 14

Three dimentional imaging of myocardial tissue stained with TMRE in the beating heart of GFP Lewis rat at 0.24 frames/second.

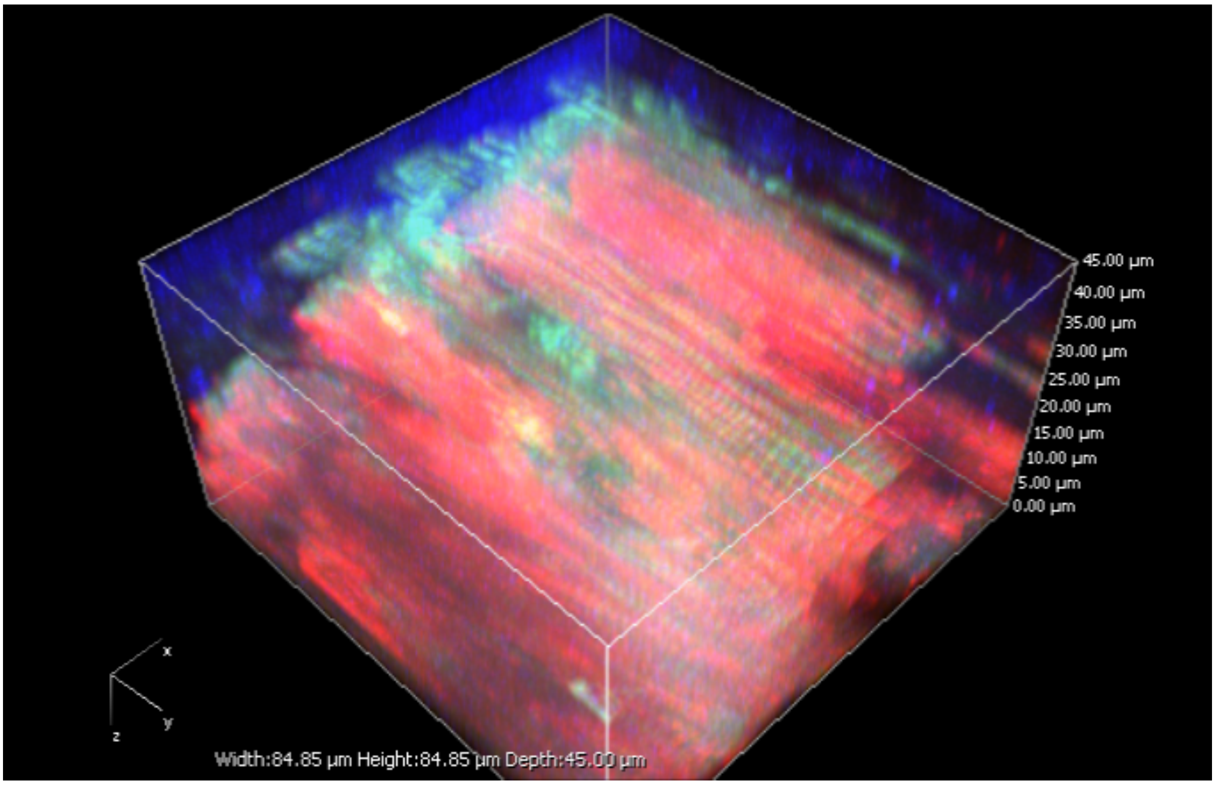

Video 1 Longitudinal two-photon imaging of the beating heart in the GFP Lewis rat. Sequential images in the same visual field were acquired every 30 minutes for 5 hours at 15 fps. (see Fig. 1c)

Videos 2 and 3. Intravital two-photon imaging of cardiac myocytes in GFP-transgenic Lewis rats (Videos 2, 3). Sequential images in the same visual field were acquired 10 min after intravenous injection of isolectin B4 (10 mg/kg) (Video 2), or 70-kDa dextran conjugated with Texas Red (10 mg/kg) (Video 3). GFP-positive myocytes can be seen in green. The capillary endothelium cells were also visualised by intravenous injection of Texas Red-dextran -conjugated isolectin B4 (red). The blood flow in the microvasculature was visualized by monitoring Texas Red-dextran fluorescence. Scale bars, 25 μm (Video 2), 10μm (Video 3). Frames were acquired consecutively at 15 frames/s. The playback speed is real-time.

Video 4. Analysis of the motion of normal cardiomyocytes was facilitated by stabilisation of the beating heart image by linear alignment, non-linear registration, and measurement of inter-pixel distances between corresponding points in the first frame and each subsequent frame of Video 2.

Video 5. Intravital two-photon imaging of cardiac myocytes in a GFP-transgenic Lewis rat. Sequential images in the same visual field were acquired 10 min after intravenous injection of TMRE (10 mg/kg) and 10 kDa Cascade Blue-conjugated dextran (60 mg/kg). A GFP-positive sarcomere can be seen in green. The distribution of mitochondria around the sarcomere was also visible. The blood flow in the microvasculature was visualized by blue-labelled dextran. Scale bar, 100 μm. Frames were acquired consecutively at 15 frames/s. The playback speed is real-time.

Videos 6 and 7. The difference in motions between high- and low-intensity patches were analysed using a video-processing program and an injured heart. Red, isolectin B4; green, GFP; blue, blue-labelled dextran; red, TMRE; scale bar, 20 μm. Frames were acquired consecutively at 15 frames/s. The playback speed is real-time.

Videos 8 and 9. Consequences of the ischaemia/reperfusion protocol. Intravital two-photon imaging of a period of ischaemia and reperfusion in a GFP-transgenic Lewis rat. Feature tracking of an arbitrary point (orange point) was used to determine the amplitude of the beating heart (Video 8). GFP-positive myocytes can be seen in green. The blood flow in the microvasculature was visualized by Texas Red-dextran. Scale bar, 100 μm. Frames were acquired consecutively at 15 frames/s. The playback speed is real-time.

Videos 10 and 11. Intravital two-photon imaging after completing a prolonged I/R protocol in a GFP-transgenic Lewis rat. Green, GFP; red, red-labelled dextran; scale bar, 100 μm. Frames were acquired consecutively at 15 frames/s. The playback speed is real time.

Video 12. Intravital two-photon imaging after completing a 30 min/30 min I/R protocol in a BMT Wistar rat. Red, red-labelled dextran; Green, GFP; Blue, Second Harmonic Generation, scale bar, 100 μm. Frames were acquired consecutively at 15 frames/s. The playback speed is real time.
